# Medical Society of the County of Albany

**Published:** 1874-09

**Authors:** F. C. Curtis

**Affiliations:** Secretary


ART. II.—*Medical Society of the County of Albany, N. Y. Semi-Monthly Meeting, May 13th,* 1874. Reported by F. C. CURTIS M. D., Secretary.

In the absence of President SWINBURNE, Dr. WM. H. CRAIG was elected President *pro tem.*

DIFFICULT CASE OF LABOR.

Dr. J. H. BLATNER reported the following case: Mrs. L., aet. 39, Multipara, robust constitution, heavy set and corpulent. Previous confinements natural and terminated without complications. In this, her ninth confinement, was attended by a mid-wife, the real "Sairy Gamp," Dickens so well describes. Labor commenced at 8 A. M., on the 25th ult., and pains continued regular and at short intervals until 2 P. M., when the membranes ruptured. At 6 P. M. he was called in great haste and was soon at the bedside of his patient, and after asking for the necessary information from the midwife, he became convinced of her total incapacity and ignorance. Making a digital examination, the fingers first came in contact with a large soft mass, lying in the right oblique diameter of the pelvis and completely filling it; in the center of this mass there was a large rent, probably measuring three inches in length. This condition of the presenting parts somewhat puzzled him, but by auscultation he found the fœtal heart above the umbilicus on the right side. He concluded with what he had previously found that the fœtus was presenting with its breech in the second position, with some abnormal condition of the anus and vulva. Ergot, after repeated trials, having no appreciable effect, was abandoned, and the patient being very much exhausted he determined if possible to bring down a foot. Owing to her exhausted condition chloroform could not be administered. Before making any attempts he sent for counsel, anticipating that he had a case of unusual severity to treat.

The consulting physician co-incided with Dr. Blatner after making a careful examination. It was now four hours since the membranes had ruptured, and the uterus had firmly contracted on the fœtus. With the greatest effort they succeeded in bringing down one foot, but with this leverage they could not bring down the trunk. After much exertion the second foot was also secured. Some little progress was now made in extracting the trunk. Every step of the operation was attended with the greatest difficulty. The forceps were applied to the head but without avail except to accelerate rotation, and only after adopting every manipulation known to them did they succeed in delivering an immense but not hydrocephalic head.

The child was of immense proportions, weighing nearly 18 pounds. The placenta and secundines soon followed, and after a few necessary precautions, the uterus contracted firmly. No hemorrhage of a serious nature followed. The patient rallied from the operation, but an hour afterwards symptoms of exhaustion manifested themselves and finally terminated in a complete state of collapse, the patient dying one hour and a half after the termination of the labor. The uterus was examined after death and found contracted and not ruptured. The placenta on the maternal side contained numerous small calcareous and ossific deposits.

Among the points of interest in the case are: 1st. The diagnostic value of auscultation as to determining the presentation of the fœtus, which in this case proved of great value. 2d. The total incapacity and ignorance of the midwife, to whose mismanagement and maltreatment much of the serious result in this case is to be attributed, the rent in the breech being undoubtedly the result of traction made by the midwife with the finger hooked in the vulva or anal opening. 3d. The calcareous deposits in the placenta. He had twice met with these deposits in the placenta and the patient was each time given to obesity. There is also a case reported in the "Southern Medical Record," where the whole attaching surface of the placenta was covered with a kind of net work of bone; in many places there were solid plates as large as a dime coin and a line in thickness. The patient was also large and compact.

Schrœder (Manual of Midwifery, p. 141) says: "Calcareous deposits of a moderate size are sometimes found in the placenta. They are found on the tip of the villi (also in the maternal tissue) and, when the deposition is more abundant, also on the pedicle. The infiltration is either more diffuse and starts from the epithelium of the villi, or what is more commonly the case it commences on the walls of the capillaries and in the vessels of the villi."

Rokitansky says, (vii p. 260): "Foreign observers have given instances of osseous deposits in or ossification of the placenta; they are gibbous, or nodular formations, which are probably developed in the placental tissue after it has been obliterated by inflammation or in the fibrinous capsule cause hemorrhage."

Dr. Lorenzo Hale spoke of a corpulent woman having had five children whom he had attended. The placenta was studded with calcareous deposits in stratified layers.

Dr. A. Van Derveer presented cases of stricture of large calibre, with the following preliminary observations:

In a few brief remarks I desire to call the attention of the Society to a class of strictures of the male urethra, which we have been too much in the habit of overlooking: It is true we can say here, as in many other branches of surgery, that our methods of examination, the instruments recently invented, &c., have enabled us to arrive at a more correct diagnosis than we were formerly in the habit of reaching. Most of us have been taught that to have a case of stricture of the urethra there must first be such a diminution or contraction presenting in the calibre of the canal, that the patient himself is first attracted to his condition. He is first to notice that his stream of urine is growing smaller, that it required a longer time for him to empty his bladder, that after a short time he finds he is compelled to make quite an effort, that the force exerted is so great as to give him much anxiety. He comes to realize the fact without consulting a physician that he has a stricture, and a careful examination only goes to confirm his opinion.

Now in contrast to some of the marked symptoms we have just noticed, we have a patient coming to us, stating that sometime within one, two, three or five years or longer, he had an attack of gonorrhœa. He may give the opinion that at the time he believed himself cured; or he may state that the doctor who attended him did not treat his case properly, and that ever since he has had an annoying gleet.

He is positive that he is passing a full stream of urine; will perhaps state that he does not have to get up at night to urinate, and will, when it is suggested, scout at the idea of his having a stricture. He is a young man of correct habits and desirous of entering the marital relation, or he may have passed on to middle life, and now, after some excess or great anxiety, he notices a condition that arouses his suspicions as to whether there is not some danger of his old disease returning. Whatever his condition in life, his case demands our most careful consideration. All of us are familiar with a class of cases who snffer from what is called gleet, and can probably each call to mind cases where, by some happy combination of medicines, as an injection or otherwise, we have been successful in stopping the discharge, (that single drop of pus, it may be, seen only in the morning,) and our reputation in consequence have been greatly enhanced.

Other cases again, apparently of like character, have taxed to our utmost our skill in the use of remedies, and while yet willing to try, we notice that our patient no longer returns, but on the contrary has gone to another doctor, perhaps in like manner to leave him after a trial of his treatment. Thus too frequently does the poor fellow travel year after year, from one physician to another. As an illustration, permit me to present the history of the following case sent me from a neighboring county.

F. C., merchant, aged 26, temperate habits, had first and only attack of gonorrhœa five years ago; discharge was profuse and very irritating; was under treatment a year and then believed himself cured. Sometime, three months after this, owing to great fatigue, he noticed a slight discharge from the urethra in the morning, and a desire to get up occasionally at night to urinate. Has consulted many physicians, but only one has ever examined his urethra and he only with No. 10 elastic bougie. This passing into the bladder with comparative ease, it was believed he had no stricture and that it was not necessary to continue its use. Has used injections of lead, zinc, nitrate silver, and various other astringents; also taken medicine internally most of the time during the past four years. I saw this patient first, March 9th, 1874. He appears strong, and says he has nothing to worry about excepting this unpleasant gleety discharge, which is very little in quantity, but he is anxious to get married and fears a return of his old gonorrhœa. Is sure that he is passing as large a stream as ever, but admits that it requires more force and a somewhat longer time than usual to empty his bladder. On examination, a No. 8 bulbous pointed bougie brings away a drop or two of gleety discharge from near the meatus, but on being re-introduced passes the entire length of the urethra, and is removed without detecting any stricture.

A No. 11 enters the meatus but meets with a decided obstruction one-third of an iuch back; this is passed after some little effort and then on into the bladder. On removing it no contraction can be detected except near the meatus, as mentioned, and here a well defined stricture, one of large calibre, can be diagnosed. The instrument is held by the stricture with sufficient firmness to hold the penis erect.

Patient is now convinced that he has a stricture, and is wi ling to submit to any treatment we think best. The stricture and meatus are freely incised with Gouley's Meatotome, and No. 16 steel sound passes into the bladder, but little hemorrhage following. Patient is taught and told to pass a large size black elastic olive pointed bougie two and three times a week, and to report again. No internal treatment ordered.

F. C. returned in three weeks, saying the discharge had ceased, and that his mind was greatly at ease concerning his old disease. Largest size bulbous bougie fails to detect any remains of stricture. Is ordered to continue the use of the elastic bougie occasionally.

Now, if we had attempted to make out a diagnosis of this man's case, according to the rules presented in many of the text-books of the present day, we should have failed. Sir Henry Thompson, with others, tells us that if No. 8, 10 or 12 elastic bougie or steel sound can be passed, the patient has no stricture. It is very evident that this can not be the case, and also, that we are not safe in our diagnosis where a gleety discharge is present, unless we have made a thorough use of the so-called bulbous bougie, in searching after the location of a stricture that may be present.

One point in our diagnosis of stricture of large calibre we are to remember, and that is, that every urethra is not of the same size. For instance, as an illustration:

A. S. treated for tight stricture, and discharged or recovered May 1st, 1873, passing No. 16 steel sound. Returned again in January, 1874, saying that while he passed a good stream, and not able to detect any of his old gleety discharge, yet of late when he had urinated and about to return his penis, he has noticed an unexpected dropping of urine, and from its soiling his garments, has become very annoying. On examination, a No. 16 steel sound, English size, passes into his bladder, causing a little pain at the meatus.

Now, it is but a few years since when all authorities would have assured us that there was nothing wrong with this man's urethra; that his difficulty must rest somewhere else; that he has no stricture. But we are inclined to examine his case somewhat more carefully. Having some trouble in introducing the large size bulbous bougie, the meatus is incised, and then we detect in the spongy portion of this man's urethra, four well defined strictures of large calibre, about one-half inch from each other, all sufficiently well marked to retain a drop or more of urine. In the treatment of this case we first tried Otis' dilating urethrotome, but succeeded best with Gouley's dilating urethrotome, and after a short time had the satisfaction of seeing this patient entirely relieved of all his troublesome symptoms, his urethra admitting the passage of Nos. 17 and 18 steel sounds, and with the largest sized bulbous bougie unable to detect any contraction of the canal.

In our examination of cases of gleet, we should ever bear in mind that the stricture of large calibre may become the tight impermeable one; that the former is treated with far better results, easier, and with less loss of life than the latter.

I believe that all cases of gleet resolve themselves into two classes—those complicated with stricture, either of large or small calibre, and those where we have the urethra presenting points of ulceration, after a severe attack of urethritis, and which, if neglected, ultimately leads to stricture.

I do not wish to take up the time of the Society in the presentation of many cases I could offer illustrating these conditions, and will present only the following, to show the good resulting from gradual dilatation, a method always at hand and easy of application, especially if it be but a short time since acute urethritis has occurred:

W. J., æt. 22, temperate habits, and has had two attacks of gonorrhœa, the last a year ago, since which time there has been a constant gleety discharge. Twice, after severe exposure, has been obliged to resort to the use of the catheter to draw off his urine, which was introduced at such times with little trouble. Examination, April 10th, 1873, with a bulbous bougie, reveals a slight stricture just back of the fossa navicularis; the remaining portion of the urethra appears healthy. Nos. 13 and 15 steel sounds were passed twice a week for a month, when the discharge entirely ceased, and has not returned up to this date, August 15th, 1873. His general health being good no internal treatment was used.

September 1, 1872. G. J., æt. 23, of temperate habits; had his first attack of gonorrhœa two and a half years ago, which was severe; recovered in three months. He was treated with copaiba only. Had a second attack eighteen months after, not severe, and was treated in same way as before. Since has had a constant discharge, a chronic gleet. No. 8 bulbous pointed bougie detects a stricture half an inch from the meatus. Gradual dilatation was practiced for three months with elastic bougies, and finally with No. 15 steel sound. Internally, tr. ferri mur., with cantharides, were given. Now, August, 1873, the discharge has entirely ceased. No. 15 is occasionally passed without difficulty.

The following case is illustrative of points of ulceration in the urethra, which we may have the good luck to locate correctly and treat by various injections, but which can be more correctly located by the endoscope, an instrument which will come into more general use as it becomes simplified in its construction:

November 1, 1872. D. D., æt. 40, intemperate, exposed to all kinds of weather, day and night. First attack of gonorrhœa eighteen years ago; treated with copaiba, injections, etc.; was four months in recovering. After this, when exposed to cold, the stream of urine becomes smaller, and for a few days subsequent there is discharge. Second attack two years ago, very severe, and attended with scalding and great pain. Treated as before, and was three months in recovering. Stream of urine became smaller, but no retention; obliged to empty his bladder during the night. Third attack four months ago. Pain acute, and occasionally passed blood with the urine. The attack yielded to copaiba and injections, but subsequently could only after frequent attempts pass a small stream. Examination shows the urethra to be in a state of ulceration for five inches, and to be uniformly contracted and roughened. The introduction of No. 2 elastic olive-pointed bougie is attended with considerable pain, and causes the part to bleed. Tincture of the chloride of iron was given with cantharides.

March 1, 1873. The occasional use of bougies up to No. 8 leaves the patient in a very comfortable condition. Discharge scant. Introduction of No. 8 bougie causes no pain. A larger size causes much pain on introduction. The use of the urethrotome or divulsor was not permitted. He uses No. 8 bougie occasionally, remaining comfortable up to the time when last seen.*

---

* Owing to lack of space a portion of the transactions for May 13th is held over until the October Number.

